# Limb-sparing surgery with latissimus dorsi flap reconstruction in extremity soft tissue sarcoma: Case series

**DOI:** 10.1016/j.ijscr.2022.107345

**Published:** 2022-06-24

**Authors:** Erwin Danil Yulian, Hana Qonita, Evelina Kodrat, Kevin Varian Marcevianto

**Affiliations:** a Division of Surgical Oncology, Department of Surgery, Dr. Cipto Mangunkusumo General Hospital, Faculty of Medicine, Universitas Indonesia, Jakarta, Indonesia; b Department of Anatomical Pathology, Dr. Cipto Mangunkusumo General Hospital, Faculty of Medicine, Universitas Indonesia, Indonesia

**Keywords:** ESTS, extremity soft tissue sarcoma, LD, latissimus dorsi, EBRT, external beam radiation therapy, IORT, intraoperative radiation therapy, Sarcoma, Soft tissue sarcoma, Limb sparing surgery, Case report

## Abstract

**Introduction and importance:**

Function-preserving and Limb-sparing surgery are now the accepted gold standard of care for Extremity Soft Tissue Sarcoma (ESTS) with the goal of surgery for STS of extremities to obtain local tumor control and minimal morbidity. Limb-sparing surgery with post-operative radiotherapy for STS results in high survival rates and local control. Adjuvant radio chemotherapy might improve distant and local recurrence in high-risk patients. Hence, we aim to present how to achieve local tumor control and minimal morbidity for high grade ESTS by conducting limb-sparing surgery combined with appropriate reconstruction and radio chemotherapy.

**Case presentation:**

We present 2 cases with high grade sarcoma that underwent limb-sparing surgery with Latissimus Dorsi (LD) flap reconstructions. Wide excisions were completed with limb-sparing surgeries for both cases with free surgical margins and LD flap reconstructions. There was no post-operative complication. Follow up examination revealed normal function of the arm. The first patient was still in remission after 2-years follow up. The second patient got pulmonary metastasis after complete resection and adjuvant radiotherapy.

**Clinical discussion:**

Limb-sparing surgery with LD flap reconstruction is able to remove the tumor completely with negative margin for the primary objective. Secondary objectives are minimizing the morbidity, maximizing postoperative body functions, as well as achieving the best cosmetic value are also achieved. LD flap is commonly easy to harvest and able to give large tissue coverage for reconstruction after surgery.

**Conclusion:**

Limb sparing surgery followed by soft-tissue reconstruction and radio chemotherapy are suitable for increasing oncologic outcome, tumor control, and limb preservation. However, inhibitions towards local recurrence and distant metastases were not guaranteed.

## Highlights

• Limb sparing surgery and radio chemotherapy maximize tumor control, oncologic outcome, and limb function
• The latissimus dorsi flap is suitable for functional and large tissue coverage reconstruction of upper extremity after limb sparing surgery
• Despite optimized local control treatment, local recurrence and distant metastases can still occur

## 1 Introduction

Being a rare group of malignancy, soft tissue sarcoma (STS) accounts for about 1 % of all adult cases and 15 % of children cases [1], [2], [4]. The National Cancer Intelligence Network (NCIN) reports that STS is approximately diagnosed in 45 per million population each year [1], [2]. In United States, it is estimated that 13.130 people are diagnosed with STS in 2020 [5]. The death toll based on this statistic time range is approximately 5.350 cases [5]. According to World Health Organization (WHO), sarcoma is classified into >50 different subtypes of STS and classified broadly into 2 categories: soft tissue and bone sarcomas [1], [2], [4]. Up to 50 % of cases occur in the extremities, where 30 % of them are in the upper limbs [6]. The major objectives are local recurrence prevention, long-term survival, function preservation, and morbidity minimization. Limb preservation surgery is currently the gold standard therapy for STS case [6]. The procedure is a wide excision including negative margin (no tumor at the margin, R0) [2], [3]. The NCCN guidelines recommend limb-sparing surgery with the goal of achieving local tumor control and minimal morbidity [4]. In the following section, two-cases study of extremity soft tissue sarcoma managed by limb preservation surgery followed by chemoradiotherapy are presented. This paper is reported in line with the PROCESS criteria [7].

## 2 Case presentation #1

**Fig. 1:**
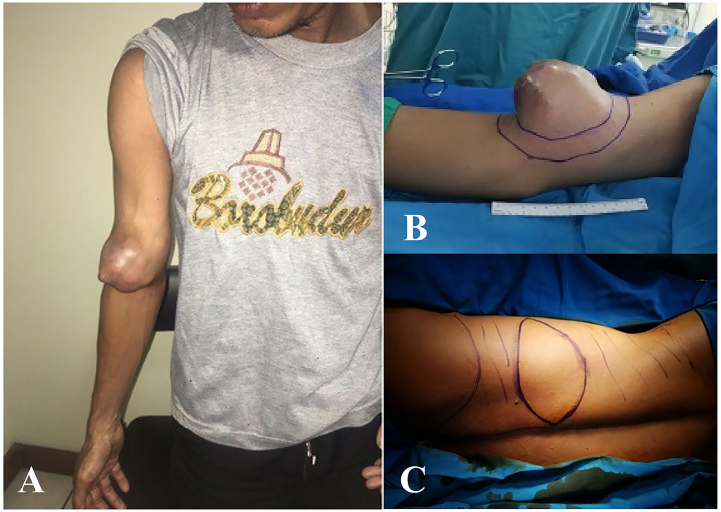
A) Irregular mass on the right upper arm, front view. B, C) Preoperative drawing of the tumor removal and the pallet of Latissimus Dorsi Muscle, dorsal view.

**Fig. 2:**
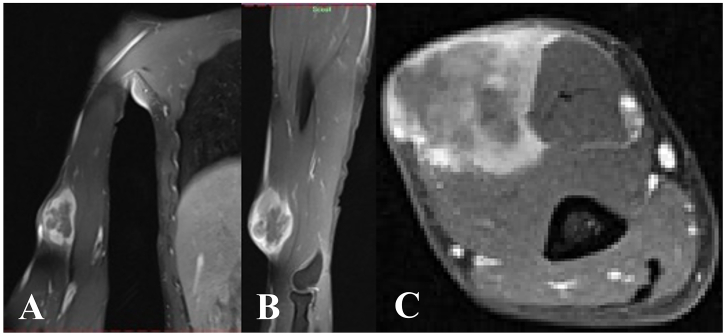
MRI of the tumor. A) Coronal view, B) Sagittal view, C) Transversal view.

A 42-year-old man was referred to our hospital with 6 months history of lump on the right upper arm. Initially, the mass size is identical to a marble. The growth of the lump was progressive and became irregular. At this point, the patient experienced pain associated with movement. He had no significant past medical history. Upon physical examination, the lump was immovable, tender, and firm on palpation with irregular surface and borders. The lump's size was10 × 10 × 10 cm and it was located 1 cm above cubiti. The skin temperature above the lump is warmer than the surrounding area. The lump surface is red and dark blue colored with a shiny surface. MRI is then done on the right upper extremity and resulted in a soft tissue tumor with necrotic tissue at ventral side of right distal humerus involving biceps brachial and brachialis muscle. There is no involvement of neurovascular on the tumor's area. Thorax X-ray and abdominal USG shows no distant metastasis. The tumor was staged as T2N0M0 based on the TNM classification (Fig. 1, Fig. 2).

**Fig. 3:**
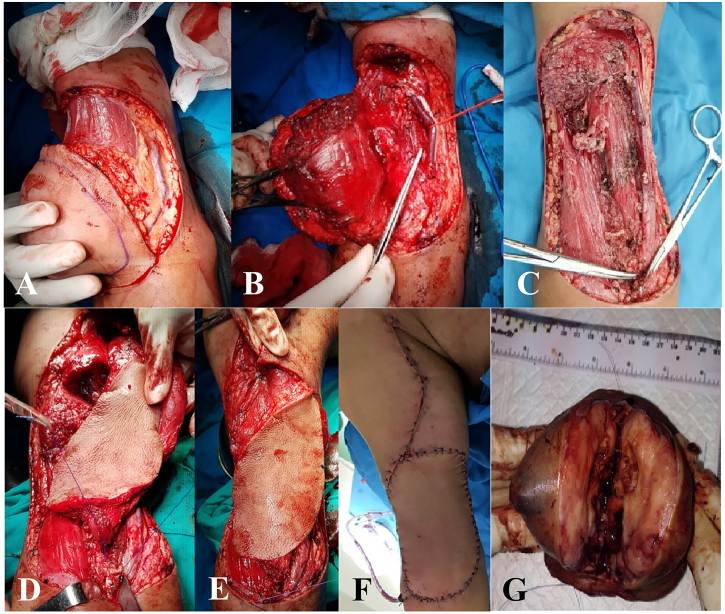
Limb sparing surgery with Latissimus Dorsi (LD) Flap reconstruction in soft tissue sarcoma. A) Incision was made 2 cm lateral from tumor borders and deepened through the fascia. B, C) Arteries, veins, and nerves were identified and preserved. C) Tumor has been removed from the arm. D, E) Flap was put into the defect area. F) Post-operative view of the arm. G) The macroscopic appearance of the tumor.

Patient was admitted for wide excision surgery and reconstruction with Latissimus Dorsi (LD) Flap. Tumor borders were marked. Incision was made 2 cm lateral from tumor borders. Incision deepened through the fascia of biceps brachial muscle. Arteries, veins, median and radial nerve were identified and preserved. The tumor did not infiltrate neurovascular. Wide excision was done. Long and short head biceps brachial muscle, part of brachialis muscle, musculocutaneous nerve and cephalic venous were elevated. Frozen section was performed intra operatively to confirm the margins were negative. Incision was done according to design and deepened to fascia of latissimus dorsi muscle. Latissimus dorsi muscle was elevated while preserving arteries, veins and thoracodorsalis nerve. Flap was put into the defect area. Distal part of latissimus dorsi was sutured to biceps brachii tendon. Medial part was sutured to long and short head of biceps brachii muscle. Surgical wound was sutured layer by layer (Fig. 3).

**Fig. 4:**
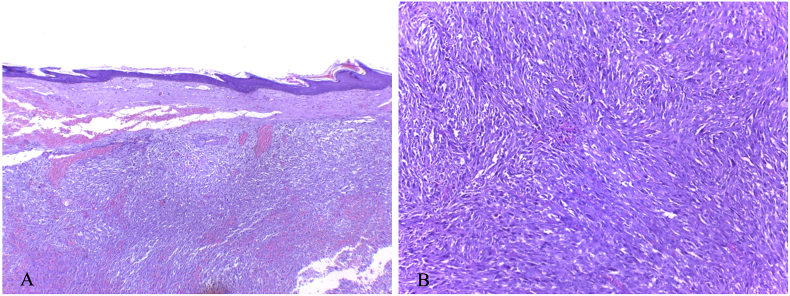
(A). The histology section showing tumor mass with marked nuclear pleomorphism that had extended to the dermis (H&E, 40×). (B). Highly atypical tumor cells arranged in fascicular pattern (H&E, 100×).

The histopathological examination revealed an undifferentiated high grade pleomorphic sarcoma with negative surgical margins (R0) (Fig. 4).

**Fig. 5:**
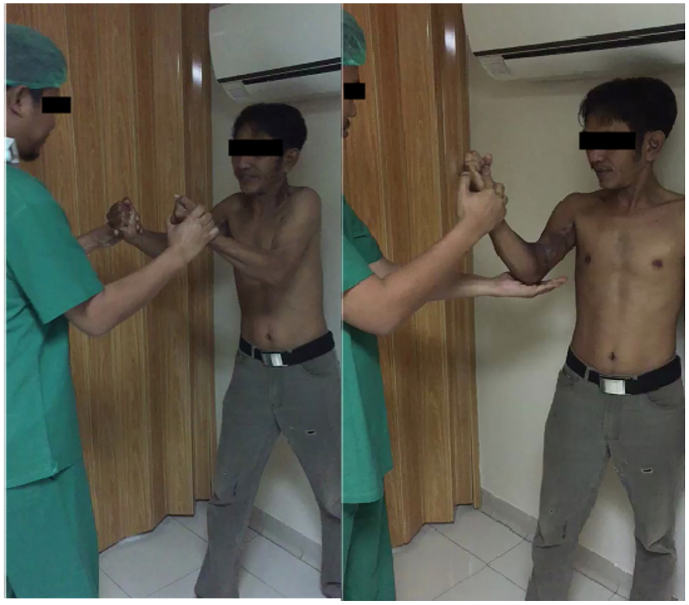
Follow up examination of muscle strength and range of motion of the elbow.

Patient was discharged on the seventh post-operative day considering there was not any postoperative complication. Follow up examination revealed normal movement and function of the arm. The patient recovered with a full strength and range of motion of his elbow (Fig. 5).

The patient then underwent radiation therapy (RT) with a dosage of 66 Gy in 33 fractions and chemotherapy with Ifosfamid, Doxorubicin and Mesna. The patient was in remission state during follow up that is done 2 years later, concluding there was no case of recurrence.

## 3 Case presentation #2

A 40-year-old man was referred to our hospital with a complaint of a lump in the upper right arm in the last 2 months. Previously, there was a lump excised 5 months ago, with anatomical pathology result of high-grade sarcoma suspect myxofibrosarcoma, rhabdomyosarcoma and hyposarcoma. The lump reappeared 3 months after the first excision. It quickly enlarged with associated intermittent pain. Patient had a history of pulmonary TB but never completed any tuberculosis treatment.

Upon general physical examination, the tumor was located in the right upper arm with the size of 4 × 5 × 4 cm, hard, fixed to muscle, with scars seen from the previous excision. There were no lumps in the regional lymph nodes. Based on non-contrast MRI, a suspected malignant soft tissue of 3.8 × 4.2 × 6 cm in the subcutis lateral to the 1/3 middle of the humeral region is found. There was a possibility of muscle infiltration to the biceps brachii muscle. No neurovascular or humeral bone involvement was seen. Based on PET SCAN, no metastases were found. A chest X-ray revealed an infiltration in the right upper lung field and the IFN-Gamma Release Assay (IGRA) resulted positive for TB infection. The patient was diagnosed with sarcoma of the right humerus T2N0M0 and pulmonary TB. The patient was given pulmonary TB treatment for 8 months.

**Fig. 6:**
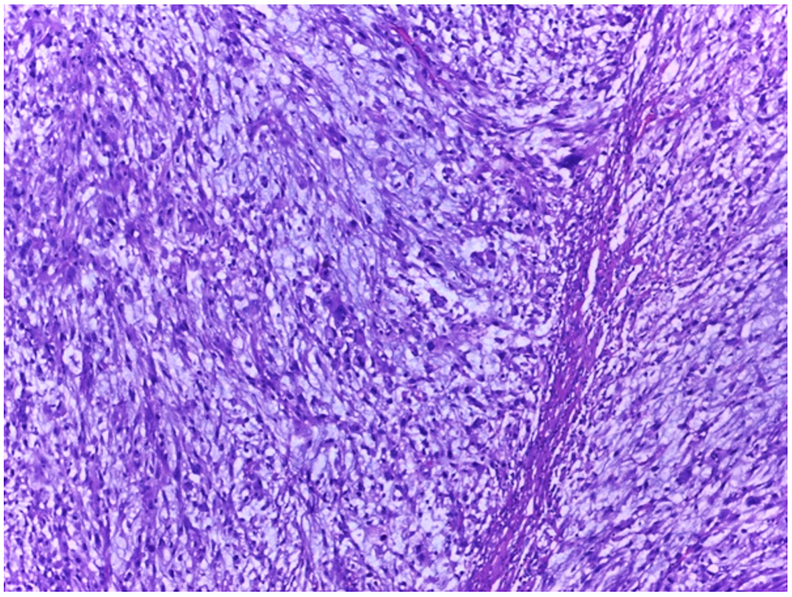
The tumor mass had myxoid stroma, highly pleomorphic cells, and elongated thin-walled blood vessel (H&E, 100×).

**Fig. 7:**
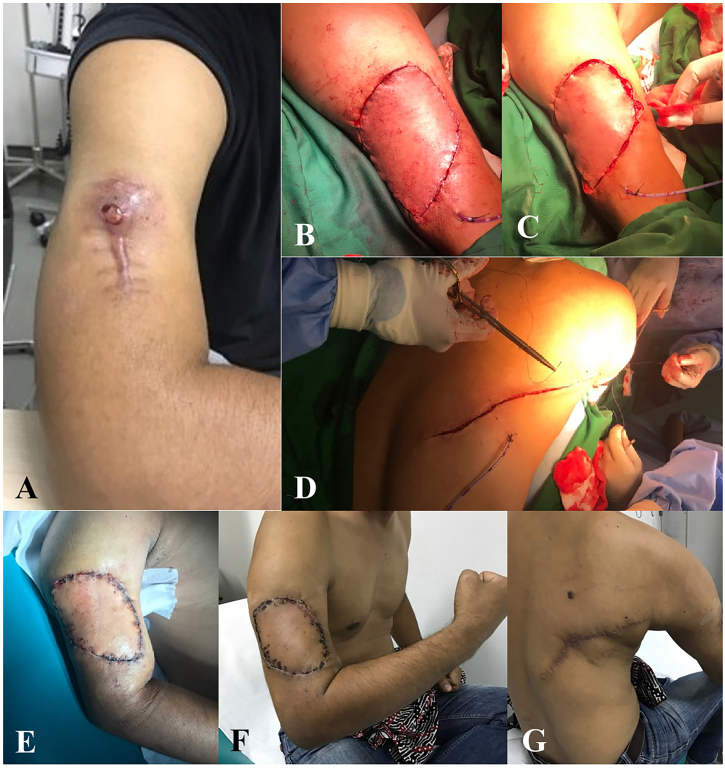
A) Tumor view on the right upper arm after previous excision. B, C, D) Limb sparing surgery with Latissimus Dorsi (LD) Flap reconstruction in soft tissue sarcoma. E, F, G) Post- operative view of the arm.

The patient underwent wide excision and latissimus dorsi (LD) flap reconstruction. An elliptical incision was made on the excision surgical site of ±2 cm. Incision was deepened through the fascia of biceps brachial muscle. The artery, vein, and radial nerve are identified and preserved. The tumor did not infiltrate the neurovascular system. Tumor with segmental biceps muscle removed. Intraoperative frozen section showing negative surgical margins. The donor was taken from latissimus dorsi muscle. The flap was transferred to the defect area. Surgical wounds were closed layer by layer. The mass was sent to pathological examination. The result showed high grade myxofibrosarcoma with a complete excision of the tumor (R0) (Fig. 6, Fig. 7).

Patient was discharged on the seventh post-operative day. No postoperative complication was noticed. Follow up examination revealed normal movement and function of the arm. He recovered a full strength and range of motion of the elbow. The patient was given post-operative radiotherapy with a total dose of 68Gy. The post-radiotherapy MRI of the right humerus with contrast resulted in neither pathological lesions nor enhancement in the soft tissues of the right brachii region. An inhomogeneous mass appeared in the lower right lung suggestive of pulmonary metastases. Then, a chest X-ray showed a large nodule in the right lateral hemithorax, and an MRI showed a thick- walled cystic mass in the right hemithorax lateral costal pleura, suggesting a loculated pleural metastasis. Fibrosis accompanied by cystic bronchiectasis in segments 1–2 of the right lung and fibrosis in segments 2–6 of the left. The patient underwent a right lung mass biopsy and FNAB. The biopsy result showed metastasis of malignant tumor with feature similar to that of the tumor in the humerus (myxofibrosarcoma, high grade). The FNAB also contained malignant tumor cells that could originate from a sarcoma.

The patient developed into pulmonary metastasis seven months after complete resection. He was consulted to a thoracic and vascular surgeon for consideration of pulmonary metastatectomy. However, there was no operative procedure from the thoracic and vascular surgery department. A chemotherapy was scheduled due to its inoperability for pulmonary metastatectomy. Unfortunately, the patient was lost to follow-up.

## 4 Discussion

We reported 2 cases with undifferentiated high grade pleomorphic sarcoma and high-grade myxofibrosarcoma which underwent limb-sparing surgery with LD Flap reconstruction. Both cases were high grade sarcoma treated with surgery and adjuvant radiotherapy. However, they have different outcomes. STS is a rare malignant mesenchyme-derived tumor which usually involves extremities. Previously, the surgical therapy of ESTS commonly is an amputation [8], [9]. Nowadays, the definitive therapy for ESTS is a radical and margin negative resection, as also for all localized sarcomas [8]. Function-preserving, limb-sparing surgery is currently the accepted standard of management [6], [8]. Overall survival for ESTS treatment has been increasing over the past years, with the 5-year survival rate up to 80 % [6]. Limb sparing surgery is indicated for (1) resectable tumor with oncological safe margins; (2) high survival possibility to justify any complex surgery; (3) the patient refusal to amputation and acceptance to the risk of local recurrence of disease due to inadequate margins; and (4) palliative patients with limb disease that can be safely operated for improving quality of life [10].

We performed limb sparing surgery for both cases referencing the gold standard care for ESTS. Based on the imaging, the tumors were resectable with oncologically safe margins, no involvement of neurovascular and safely operated to improve quality of both patient's life. There were no regional and distant metastases. The objective to preserve a limb and its function after surgery might need complex soft-tissue & neurovascular reconstruction. This might require rotational fasciocutaneous or myocutaneous flap, full-thickness skin graft, free flap, split-thickness skin graft, pedicled muscle flap, nerve interposition, and vascular graft [8]. Soft-tissue reconstruction can be achieved by using musculocutaneous, muscular, and cutaneous pedicled flaps on the donor surface [6]. The latissimus dorsi (LD) pedicled flap is usually chosen for the functional and soft-tissue coverage reconstructions of upper extremity [9]. The LD flap is also can be considered for any extensive defect due to a resection. This is because it is commonly easy to harvest and able to give large tissue coverage [6]. Moreover, the function of the flap is supported with minimal donor site morbidity [6], [9]. Complication following limb sparing surgery may be the risk of infection correlated with the duration of surgery, neovascular injury, devascularization of the soft tissue flaps and fractures [10]. In both cases, we performed limb-sparing surgeries with LD flap reconstructions since they were easy to harvest and able provide large tissue coverage to provide restoration of upper-extremity function with minimal donor site morbidity. On post-operative follow up, both patients have no postoperative complication and show a full range of motion and strength of elbow. Limb-sparing surgeries with LD flap reconstructions that were completed for the two patients in this report have been able to achieve the main objective of the surgery which is to remove the tumor completely and healthy tissue peritumor (surgical margin R0). Secondary objective such as minimizing the morbidity, maximizing bodily functions post- surgery, as well as achieving the best cosmetic value were also achieved.

Integration of multidisciplinary approach involving surgery with neoadjuvant or adjuvant radiation therapy (RT) and/or chemotherapy is able to provide local control in approximately 90 % of cases and result in significant enhancement on disease-free survival [6], [8]. The abilities of adjuvant therapy include (1) local tumor necrosis induction; (2) tumor size decrement; and (3) building a peritumoral “rind” of fibrous capsule [10]. RT is able to reduce recurrence rate yet preserving the limb and its function after a margin-negative resection [7]. Preoperative or postoperative External Beam RT (EBRT) has been reported to enhance the local control in a high-grade lesion. Large sized stage II or III extremity yet resectable tumors which are at high risk for local recurrence and metastases are indication for preoperative and postoperative RT. Surgery followed by radiotherapy with/without chemotherapy is the primary therapy for stage IIIA or IIIB cases which are resectable with acceptable functional results [4]. NCCN recommended EBRT for patient without prior preoperative radiotherapy. The alternatives include EBRT (50Gy irrespective of surgical margins in 1.8–2.0 Gy per fraction), Intraoperative RT (IORT) (10–16 Gy followed by 50 Gy EBRT), or brachytherapy. For patients with postoperative EBRT, it is recommended that they need additional EBRT boost based on the margin status (20–26 Gy for grossly positive margins, 16–18 Gy for microscopic residual, and 10–16 Gy for negative surgical margin,) [3], [4]. A retrospective study of 983 high-grade STS patients underwent radical limb-sparing surgery with or without postoperative EBRT. The 3-year OS for tumor >5 cm was 73.4 % in the postoperative radiation group and 55.6 % in the surgery alone group [11]. Limb-sparing surgery with post-operative radiotherapy results in excellent local control and high survival rates for STS patients [12]. In this report, both patients received post-operative RT based on the guidelines and relevant studies, with dosage 66 Gy in the first case and 68 Gy in the second case. Radiotherapy is done to provide optimized local control, reduce risk of local recurrence and increased high survival rates. It is necessary to provide adequate reconstruction following radiation therapy, which is usually at high doses (>50 Gy). Neoadjuvant irradiation tends to elevate disunion or delayed cicatrization risks, especially if a wound is closed under tension. Hence, soft tissue reconstruction is able to contribute in effective wound healing. Following an excision for limb STS in irradiated patients, utilization of resistant flaps with strong vascularization results in faster wound healing, revision and secondary covering procedures reductions, and faster hospital stay [13].

Adjuvant and neoadjuvant chemotherapy are still subject to controversies for the treatment of STS, except Ewing sarcoma and rhabdomyosarcoma [8]. The benefits resulted from postoperative chemotherapy for stage II or III diseases are included as a stage 2B recommendation because there are limited yet conflicting data [4]. In the presence of negative results from the largest studies, there are available data from smaller studies suggesting adjuvant chemotherapy might improve the risk distant and local recurrence in high-risk patients (high-grade, deep >5 cm tumor) [2], [3]. In the first case, the patient received adjuvant chemotherapy with with Ifosfamid, Doxorubicin and Mesna since the patient's tumor >5 cm and histologically showed a high-grade sarcoma. With the consideration from multidisciplinary team, we had given adjuvant chemotherapy to the patient to reduce distant and local recurrence that might improve overall survival.

Instead of optimal local treatment and local control, 20 % - 30 % of STS develop metastases. The overall 5-year survival of metastatic STS patients has not improved in the last decades [14]. Pulmonary metastases occurred in 50 % of high-grade STS, usually in the first 2 years after the primary tumor diagnosis [15]. Metachronous, resectable lung metastases without any extrapulmonary disease are treated with surgery, if complete excision is feasible [3]. Due to no data available to support the optimal management of metastatic disease, according to the NCCN, it is recommended to refer the case to a medical oncologist with adequate experience and knowledge in the STS treatment [4].

A retrospective study examined 94 patients with intermediate or high- grade localized ESTS got radical resection and RT. 32 % of them experienced recurrence after 60 months median of follow up [4]. In another retrospective study of 1580 patient with STS, 130 patients (9 %) were diagnosed with pulmonary metastasis as first systemic relapse after local treatment. The 1-, 5- and 10-year OS were 63 %, 24 % and 15 %, respectively [16]. The most common subtypes developing reccurence and metastasis were leiomyosarcoma (22.4 %), UPS/MFH (22.4 %), synovial sarcoma (10.2 %) and myxofibrosarcoma (10.2 %) [15]. The largest North American cohort of myxofibrosarcoma (MFS) reported that the 5-year OS of 69 high-grade patients who underwent surgical resection was 53 %. Distant metastatic disease occurred in 14 patient (20 %) and ten patient (15 %) developed lung metastasis. Myxofibrosarcoma has a propensity for local recurrence due to its infiltrative growth. 50 % of recurrences showed higher-grade histology and greater metastatic potential than the primary lesion [17]. In our case, the histological finding of the second case was a myxofibrosarcoma. Patient have already undergone excision before being referred to our center, however the tumor recurred 3 months post excision. Even though the surgery with post-operative RT had been done to achieve optimal control, lung metastasis still occured. Hence, another additional treatment might be needed to increase the survival of such cases.

## 5 Conclusion

Limb sparing surgery followed by soft-tissue reconstruction and radio chemotherapy is suitable for maximizing tumor control, oncologic outcome, and limb function for soft tissue sarcoma. Moreover, an inhibition towards local recurrence and distant metastases were not guaranteed.

## Presentation at a meeting

None declared.

## Declaration of competing interest

The authors declare no conflict of interests.
